# Nervous System in Disease

**Published:** 1869-10

**Authors:** 


### Nervous System in Disease.

Dr. Louis A. Tourtelott read before the Oneida County Medical Society, at its last meeting, Oct. 11, a carefully prepared paper entitled "The Nervous System in Disease," of which the following is an abstract:

### THE NERVOUS SYSTEM IN DISEASE.

He began by saying that the time had long gone by when medical service was built upon any single theory, and its practice governed by rules derived from that theory. Homœpathy now stood alone as a pretended philosophy of disease, and a system of practice strictly deduced from theoretical principles. It was because legitimate medicine rejected any such narrow and fictitious bases that it could not accept the name of allopathy on any other exclusive term. But he did not intend to state that medicine had been for more than a century without accredited principles. It was, in fact, the heir of all the ages in this respect. He then notices briefly the principal theories which have had their rise and fall in the history of medicine, and traced their influence upon the present doctrines and practice. The theories of humoralism arose more than eighteen hundred years ago; and bleeding, their chief contribution to practice, had but just now been discarded. The rise of natural philosophy had given origin to physical theories of disease, and to new modes and new instruments of physical exploration. Again, the brilliant discoveries in chemistry had given chemical principles the lead in theory, and new methods of analysis, and new chemical compounds were added to the medicine.

But it was to a later direction of medical thought that he wished to call their attention. The relations of the nervous system to physiology and pathology were now being closely studied, and there was a constantly growing belief in their importance. Many symptoms and many disorders which before had been differently classified,were now considered nervous. Several of these were cited, and important papers in medical journals were referred to in proof of the change going on.

The causes of this tendency to attribute more and more to the functions of the nervous system, were then traced. The chief cause was found in the great advance lately made in the minute anatomy and physiology of the nervous system. This was illustrated at length, from the discoveries of Brown, Sequard and others. Another cause was the more definite idea attained of the vital force and its relations with the physical forces. It had been demonstrated that the grand central function of the human organism, the functions of nutrition, to which all the others are merely accessory, was purely vital. Thence proceeded the modern idea of disease as a deficiency simply of vital force.

The changes in medical theory and practice in accordance with these views, were then described. It was admitted that the most of the numerous remedies which had been devised to act chemically upon the system, had not answered their purpose. This was because the merely chemical changes in the human body were non-essential, or at least secondary. But such remedies had been too often excessively used. On some fanciful idea of their chemical action, the most deadly poisons had been recklessly experimented with, to the great prejudice of health. This point was illustrated at length, by reference to several popular remedies. The fallacy of the theories which had suggested them, and their baneful or negative effects, were pointed out. The more satisfactory explanation of the action of certain valuable medicines afforded by the nerve theories was also given. Finally, a resume of the views set forth in the paper were as follows:

1. The nervous system, being the special organ of all that is peculiar and most essential to the human organism,—its purely vital actions—should have the highest place in medical study aud observation.

2. Imperfect and perverted nutrition—changes in the primary cells, being the starting point of disease, the main efforts of the physician should be to supply the essential condition of this process.

3. Chemical and mechanical views of disease, having led to the excessive use of drugs whose chief quality is to depress the powers of the nervous system, and to delay or prevent vital changes, deserve the severest criticism.

4. All substances which are essentially devitalizing, and those which are purely stimulant only, should be cautiously employed, and for the shortest possible periods ot time.

5. As in the language of an American author, "the most unwarrantable of all experiments are those involving human health," the free experimenting with poisonous drugs in general practice, should be strongly condemned.—*Utica Hearald.*

